# Immunogenicity and protective efficacy of *Salmonella enterica* serovar Pullorum pathogenicity island 2 mutant as a live attenuated vaccine candidate

**DOI:** 10.1186/s12917-015-0497-3

**Published:** 2015-07-24

**Authors:** Junlei Yin, Zhao Cheng, Lijuan Xu, Qiuchun Li, Shizhong Geng, Zhiming Pan, Xinan Jiao

**Affiliations:** Jiangsu Key Laboratory of Zoonosis, Jiangsu Co-innovation Center for Prevention and Control of Important Animal Infectious Diseases and Zoonoses, Yangzhou University, Yangzhou, Jiangsu 225009 P R China

**Keywords:** *Salmonella enterica* serovar Pullorum, Pullorum disease, *Salmonella* pathogenicity island 2, live attenuated vaccine

## Abstract

**Background:**

*Salmonella enterica* serovar Pullorum (*S.* Pullorum) causes Pullorum disease (PD), a severe systemic disease of poultry and results in considerable economic losses in developing countries. In order to develop a safe and immunogenic vaccine, the immunogenicity and protective efficacy of S06004ΔSPI2, a *Salmonella* pathogenicity island 2 (SPI2) deleted mutant of *S.* Pullorum was evaluated in 2-day old chickens.

**Results:**

Single intramuscular vaccination with S06004ΔSPI2 (2 × 10^7^ CFU) of chickens revealed no differences in body weight or clinical symptoms compared to control group. S06004ΔSPI2 bacteria can colonize and persistent in liver and spleen of vaccinated chickens approximately 14 days, and specific humoral and cellular immune responses were significantly induced. Vaccination of chickens offered efficient protection against *S*. Pullorum strain S06004 and *S*. Gallinarum strain SG9 challenge, respectively, at 10 days post vaccination (dpv) based on mortality and clinical symptoms compared to control group.

**Conclusions:**

These findings suggest that S06004ΔSPI2 appears to be a highly immunogenic and efficient live attenuated vaccine candidate.

## Background

*Salmonella enterica* serovar Pullorum (*S.* Pullorum) is the causative agent of Pullorum disease (PD), an acute systemic disease that results in high morbidity and mortality in young chicks and a loss of weight, decreased fertility and hatchability, lesions, diarrhea and abnormalities of the reproductive tract in infected adults, it can be transmitted vertically to chicks through eggs [1]. This disease remains a big threat of restricting the growth of the poultry industry in developing countries [2]. As a close relative of *S.* Pullorum, *Salmonella enterica* serovar Gallinarum (*S.* Gallinarum) causes Fowl typhoid (FT), a severe systemic disease with significant morbidity and mortality in poultry in many countries [2–5].

Vaccination is an effective strategy for the control of *Salmonella* infections, both humoral and cellular immunity are required for ideal *Salmonella* vaccines [6]. Live vaccines offer greater protection than killed vaccines because higher cellular immune response could be induced, it is important for clearance of *Salmonella* infections [6].

As an indispensable virulence determinant associated with the systemic infections, *Salmonella* pathogenicity island 2 (SPI2) can encode type III secretion system 2 (T3SS2), which is induced after invasion, and the T3SS2 secreted effectors are essential for *Salmonella* to survive and replicate inside various cell types [7, 8]. There are some papers on the vaccine potential of *S.* Enteritidis, *S.* Typhimurium and *S.* Typhi mutants with deletion of SPI2 or other key genes located within the pathogenicity island display decreased virulence in poultry, pigs, cattle, mice, and humans [9–14]. Therefore, in order to determine whether the SPI2 mutant strain of *S.* Pullorum has the vaccine potential, we evaluated the immunogenicity and protective efficacy of S06004ΔSPI2 in susceptible HY-line white chickens. Our results showed that intramuscular vaccination with S06004ΔSPI2 provides efficient protection against challenges with *S.* Pullorum and *S.* Gallinarum.

## Methods

### Experimental animals

The animal experiments were conducted with the approval of the Animal Care and Ethics Committee of Yangzhou University. HY-line white chicken eggs were hatched and the chickens were detected for freedom from any clinical signs of enteric disease and negative for *Salmonella*. Two-day old chickens were used in this study and given antibiotic–free food and water throughout the experimental period.

### Bacterial strains

*S.* Pullorum S06004 (accession No. CP006575.1), a nalidixic acid-resistant (Nal^r^) clinical isolate obtained from chickens with Pullorum disease in the Jiangsu Province of China in 2006 [15], and the virulent wild type *S.* Gallinarum strain SG9 (Nal^r^), supplied by Dr. Barrow [16], were used as challenge strains. S06004ΔSPI2 (Nal^r^, the whole SPI2 (~40 kb) deleted mutant of *S*. Pullorum S06004), constructed using the one-step inactivation method described by Datsenko and Wanner [17, 18], was used as the vaccine candidate for this study. Bacterial strains were stored as frozen cultures in Luria-Bertani (LB) broth with 20 % glycerol at −70 °C before use. LB broth, LB solid (15 g/L agar) and XLT4 (Difco) agar were used for culturing bacteria at 37 °C. The media were supplemented with Nal (40 μg/ml) as required.

### Bacterial inoculation in chickens

One hundred 2-day old chickens were randomly assigned to 2 groups: vaccinated group (*n* = 45) and control group (*n* = 55). The vaccinated group was intramuscularly immunized with 2 × 10^7^ CFU S06004ΔSPI2 in 100 μl phosphate buffered saline (PBS), while control group was unimmunized and only received equal amounts PBS.

### Changes of body weight and clinical symptoms after vaccination

Body weights of these chickens were measured at 5, 12 and 19 days post vaccination (dpv), and they were monitored for 19 days for clinical signs of disease, which included anorexia, diarrhea and depression, etc.

### Bacterial persistence and clearance from internal organs

Liver and spleen samples of five chickens from each group were aseptically collected at 5, 7, 10, 14 and 21 dpv for bacterial recovery. Then they were weighed and suspended in 1 ml PBS and homogenized individually. Homogenates (100 μl) of different dilutions were inoculated on XLT4 agar (containing 40 μg/ml Nal) for enumeration and incubated for 20 h at 37 °C. The bacterial number in the sample was counted and expressed as log10 CFU/g, negative samples were indicated as 0 CFU/g.

### Immune responses induced by the vaccine strain

Humoral immune responses were evaluated through determination of Specific antibody IgG levels by Enzyme**-**linked immunosorbent assay (ELISA), using heat-killed whole *S.* Pullorum bacteria as coating antigen as previously described [19]. Serum samples were collected from five chickens of each group at 3, 7, 14 and 21 dpv, and diluted 1:50 to be used as the primary antibody. The secondary antibody was Horseradish peroxidase (HRP)-conjugated rabbit anti-chicken IgG (1:10,000 dilution). The bound HRP activity was determined using o-phenylenediamine dihydrochloride (Sigma), and the OD_492_ was determined with an ELISA reader after the reactions were stopped by 2 M H_2_SO_4_.

Cellular immune responses were evaluated by the peripheral mononuclear cell proliferation assay as previously described [20, 21]. Soluble antigen was prepared from the wild type *S.* Pullorum strain S06004. Peripheral lymphocytes were separated from blood of five birds per group using the Histopaque®-1077 (Sigma) at 7, 14 and 21 dpv. After trypan blue dye exclusion testing, a viable mononuclear cell suspension (100 μl) at 1 × 10^6^ CFU/ml in RPMI-1640 medium with 10 % fetal calf serum, 2 mM L-glutamine, 50 U/ml of penicillin and 50 μg/ml of streptomycin was incubated in triplicate in 96-well tissue culture plates with 50 μl of medium alone or medium containing 4 μg/ml of soluble antigen at 41 °C (in a humidified 5 % CO_2_ atmosphere for 48 h). The proliferation of stimulated lymphocytes was measured using adenosine triphosphate (ATP) bioluminescence with the ViaLight® Plus Kit (Lonza Rockland, ME, USA). The blastogenic response against soluble antigen was expressed as the mean stimulation index (SI) as previously described [20].

### Evaluation of immune protection

Protective efficacy of S06004ΔSPI2 against challenges with *S.* Pullorum and *S.* Gallinarum were assessed, based on survival rates and clinical symptoms (including anorexia, diarrhea, depression, high morbidity and mortality). At 10 dpv, twenty chickens from vaccinated group were randomly divided into two groups of 10 animals (group A and C), thirty chickens from control group were randomly divided into three groups of 10 animals (group B, D and E). Group A and B were challenged intramuscularly with 2 × 10^9^ CFU S06004 in 100 μl of PBS. Groups C and D received equal amounts of SG9. Group E only received 100 μl PBS. The surviving birds were counted at 21 days post challenge, and clinical symptoms were recorded every day from 1–35 dpv.

### Statistical analysis

All data were expressed as mean ± standard error of the mean (SEM) values unless otherwise specified and analyzed with GraphPad Prism. P values less than 0.05 were considered significant when using one-way analysis of variance (ANOVA).

## Results

### Changes of body weight and clinical symptoms after vaccination

After vaccination with S06004ΔSPI2, the mean body weight of each chicken in vaccinated group and control group at 5, 12 and 19 dpv were shown in Table 1. No significant differences and no clinical signs (anorexia, diarrhea and depression) were observed between the two groups.

**Table 1:** Mean body weights of chickens after vaccination. The vaccinated group was intramuscularly immunized with 2 × 10^7^ CFU S06004ΔSPI2 in 2-day old chickens, and control group received 100 μl PBS

Group	Mean body weight per chicken at dpv (g)		
	5	12	19
vaccinated	65.416 ± 0.418	113.878 ± 0.493	186.583 ± 0.716
Control	64.592 ± 0.782	114.618 ± 0.795	187.171 ± 0.385

There were no significant differences between groups at any time point (*P* > 0.05)

### Bacterial persistence and clearance in internal organs

**Fig. 1:**
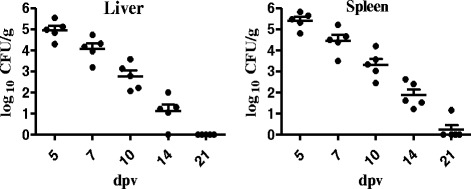
Bacterial recovery from liver and spleen of the vaccinated chickens. The vaccinated group was intramuscularly immunized with 2 × 10^7^ CFU S06004ΔSPI2 in 2-day old chickens, and control group received 100 μl PBS. Values represent the mean ± SEM log_10_ CFU/g. All liver and spleen samples of control group were negative

All liver and spleen samples of control group were negative for *Salmonella* recovery. As shown in Fig. 1, the considerably decreased bacterial counts of vaccinated group were continuously observed through to 21 dpv in both liver and spleen, but S06004ΔSPI2 bacteria can colonize and persistent in liver and spleen of vaccinated chickens approximately 14 days. Only one spleen sample was positive and no liver sample was positive at 21 dpv.

### Humoral and cellular immune responses

**Fig. 2:**
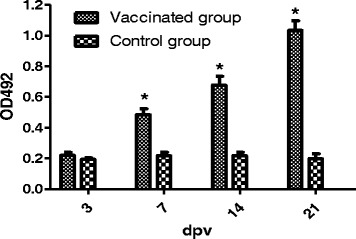
Determination of serum IgG levels. Vaccinated group and control group refer to Fig. 1. Values represent the mean ± SEM. *Significant difference compared to the control group, *P* < 0.05

Humoral immune responses were evaluated by measuring specific serum IgG levels at 3, 7, 14 and 21 dpv using ELISA. The mean OD_492_ values of vaccinated group were 0.221 ± 0.019, 0.484 ± 0.039, 0.678 ± 0.056 and 1.032 ± 0.064 at 3, 7, 14 and 21 dpv, respectively (Fig. 2). The chickens in vaccinated group had significantly higher serum IgG levels than those in control group at 7, 14 and 21 dpv. The considerably elevated serum IgG levels of vaccinated group were continuously observed through to 21 dpv.

**Fig. 3:**
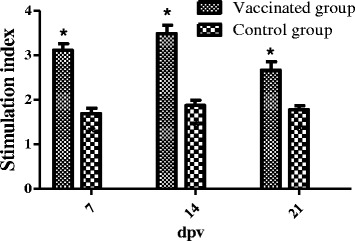
Stimulation index (SI) of chicken lymphocyte samples determined by peripheral lymphocyte proliferation assay using soluble antigen. Vaccinated group and control group refer to Fig. 1. Values represent the mean ± SEM. *Significant difference compared to the control group, *P* < 0.05

Cellular immune responses were examined by the peripheral mononuclear cell proliferation assay. The mean SI values of vaccinated group were 3.124 ± 0.138, 3.495 ± 0.188 and 2.667 ± 0.189 at 7, 14 and 21 dpv, respectively (Fig. 3). All tested chickens in vaccinated group revealed considerably elevated SI values compared to control group, and the significantly elevated SI values was continuously observed at 14 dpv, but was reduced at 21 dpv.

### Immune protection

The percent survival of chickens which had been vaccinated intramuscularly with *S.* Pullorum mutant S06004ΔSPI2 followed by challenge with the parent *S*. Pullorum strain S06004 or *S*. Gallinarum strain SG9 at 10 dpv was shown in Table 2. One immunized chicken died, whereas nine chickens died in control group B after challenged with S06004. Three immunized chickens died, whereas all ten chickens died in control group D after challenged with SG9. The clinical symptoms (high morbidity and mortality, anorexia, diarrhea, depression) of group A and C were slight and temporary after challenged compared to group E, and the chickens had recovered by 3–7 days post challenge; but these clinical symptoms were observed in group B and D. S06004ΔSPI2 conferred effective protection.

**Table 2:** Protective efficacy of S06004ΔSPI2. Group A and C were intramuscularly immunized with 2 × 10^7^ CFU S06004ΔSPI2 in 2-day old chickens, group B, D and E were nonimmunized. At 10 dpv, group A–D were challenged

Group	Vaccination	Number	Challenge			Survivors/Total	Survival rate (%)
	Strain		Strain	Route	Dose (CFU)		
A	S06004△SPI2	10	S06004	intramuscularly	2 × 10^9^	9/10	90*
B	PBS	10	S06004	intramuscularly	2 × 10^9^	1/10	10
C	S06004△SPI2	10	SG9	intramuscularly	2 × 10^9^	7/10	70*
D	PBS	10	SG9	intramuscularly	2 × 10^9^	0/10	0
E	PBS	10	—	—	—	10/10	100

*P < 0.05 for comparison of group A with group B, and group C with group D

## Discussion

In this work, we evaluated the immunogenicity and protective efficacy of a *Salmonella* pathogenicity island 2 (SPI2) deleted mutant of *S.* Pullorum (S06004ΔSPI2) to serve as a live vaccine against PD and FT in susceptible HY-line white chickens on the basis of changes of body weight and clinical symptoms, bacterial persistence and clearance, humoral and cellular immune responses, and protective efficiency.

In order to evaluate the effects of S06004ΔSPI2 on growth performance in chickens, we recorded the body weight increases and observed the clinical symptoms after intramuscular vaccination. Our results showed that S06004ΔSPI2 has almost no side effects on growth performance in chickens. T3SS2 encoded by SPI2 is essential for *Salmonella* colonization and persistence in host. With the absence of a functional T3SS2, *Salmonella* is cleared more rapidly than the parental wild type strain from the host, and some studies have failed to isolate SPI2 mutants from liver and spleen after oral inoculation [5, 16, 22]. Here, our results showed that S06004ΔSPI2 can colonize and persist in liver and spleen of vaccinated chickens approximately 14 days, this may be related to the breed of chicken, the routes of vaccination and the dose of inoculation.

Specific humoral and cellular immune responses induced by the live attenuated vaccines of *Salmonella* are crucial for the natural host [6]. To investigate the specific humoral immune responses imparted by the candidate, we examined the specific serum IgG antibody level by indirect ELISA, there was a strong specific serum IgG level in vaccinated chickens, and the antibodies were detectable at 7 dpv. The vaccinated chickens showed significantly elevated IgG levels compared to non-immunized chickens. *S.* Enteritidis SPI2 mutant can also induce significant increase of antibodies in chickens [9]. Cellular immune responses play a central role in protection against *Salmonella* challenge, because *Salmonella* are facultative intracellular pathogens [23]. We further evaluated the cellular immune responses imparted by the candidate in chickens using the peripheral lymphocyte proliferation assay. In the present study, a significantly elevated cellular immune response was clearly observed in chickens immunized with S06004ΔSPI2, but the significantly elevated SI value was decreased at 21 dpv, it is related to the restricted colonization of bacteria in internal organs [24, 25]. Taken together, the specific humoral and cellular immune responses were clearly observed in the vaccinated chickens in this study.

Several previous reports have shown that live attenuated *Salmonella* vaccines can confer effective cross-protection to different pathogenic *Salmonella* serovars [26, 27]. Here, we evaluated the protective efficacy of the candidate vaccine against challenge intramuscularly with *S*. Pullorum and *S*. Gallinarum, respectively, based on survival rates and clinical symptoms in HY-line white chickens. The survival rates were 90 % and 70 % following respective challenge with *S*. Pullorum and *S*. Gallinarum in vaccinated chickens; but in the control groups, the survival rates were 10 % and 0, respectively. The light and temporary clinical symptoms of vaccinated chickens (group A and C) had recovered by 3–7 days post-challenge. Recently, our results also showed that S06004ΔSPI2 can be used as a live attenuated oral vaccine [28]. Overall, these results showed that the candidate vaccine S06004ΔSPI2 can afford effective protection for acute systemic PD and FT infection.

## Conclusions

Our present work demonstrated that the vaccination of susceptible chickens with the candidate vaccine S06004ΔSPI2 conferred development of acquired immunity and efficient protection for the experimental systemic PD and FT infection. Taken together, the SPI2 mutant strain of *S.* Pullorum has the potential of being used as a safe, novel, highly immunogenic vaccine against PD and FT.

## Abbreviations

*S.* Pullorum: *Salmonella enterica* serovar Pullorum
*S.* Gallinarum: *Salmonella enterica* serovar Gallinarum
PD: Pullorum disease
FT: Fowl typhoid
SPI2: *Salmonella* pathogenicity island 2
T3SS2: Type III secretion system 2
Dpv: Days post vaccination
LB: Luria-Bertani
PBS: Phosphate buffered saline
ELISA: Enzyme**-**linked immunosorbent assay
SI: Stimulation index
